# S5-5 Developing an individual-based advice for sports participation and physical activity in children using the concept of Physical Literacy

**DOI:** 10.1093/eurpub/ckad133.027

**Published:** 2023-09-11

**Authors:** Amika Singh, Sanne Veldman

**Affiliations:** Mulier Instituut, Utrecht, The Netherlands; Mulier Instituut, Utrecht, The Netherlands

## Abstract

**Purpose:**

Practitioners, researchers and policy makers have been looking for ways to change the negative trend observed in physical activity (PA) behaviour in children. An individual PA advice for children, based on the concept of physical literacy (PL), might offer an important avenue to support and motivate children to be physically active in a way that fits their needs and skills. Due to the holistic approach of PL, this concept is suitable for the development of an individual PA advice to motivate children to be physically active for life. Therefore, we aim to develop a feasible tool to provide children aged 4 to 12 years with an individual PA advice in the Dutch school setting.

**Method:**

In this project, experts on the different areas of PL collaborate to create a tool feasible for implementation in the Dutch primary school setting. Experts will comprise children, parents, practitioners, researchers and policy makers.

First, available measurement instruments on (elements of) PL will be reviewed. This will help us determine what instruments are best suitable to be used in the Dutch setting to create ‘input’ for a tool. Secondly, we will examine how data from these instruments can be harmonized to create an individual PA advice. This step will involve close collaborations with ICT while adhering to privacy rules and regulations. During this step we will also examine how the translation from ‘input’ to ‘output’, an individual PA advice, might have to differ for different age groups. Thirdly, we will develop a plan for the implementation in the Dutch setting and educational context. This involves feasibility in terms of finances as well as feasibility from a practical point of view.

**Conclusion:**

By creating a tool to develop an individual PA advice, we hope to support and motivate children and parents to engage in PA that will last a life time. Throughout the process, relevant stakeholders will be involved in any step needed to ensure acceptability, feasibility and sustainability.

**Funding:**

This project is funded by the Dutch Ministery of Health, Welfare, and Sports.

